# Subhepatic Abscess Unmasking the Silent Gastric and Pulmonary Sarcoidosis

**DOI:** 10.7759/cureus.16957

**Published:** 2021-08-06

**Authors:** Brinda Basida, Maryam B Haider, Anusha Bapatla, Nirav Zalavadiya, Sana Iqbal

**Affiliations:** 1 Internal Medicine, Detroit Medical Center Sinai-Grace Hospital, Detroit, USA; 2 Internal Medicine, Detroit Medical Center/Wayne State University Sinai-Grace Hospital, Detroit, USA

**Keywords:** sarcoidosis, gastric sarcoidosis, pulmonary nodules, non necrotizing granulomas, gastric biopsy

## Abstract

Sarcoidosis is a non-necrotizing granulomatous disease of unknown etiology presenting with variable systemic manifestations. Lung involvement is the most common initial presentation of sarcoidosis. Rarely, patients can present with initial non-pulmonary symptoms. Asymptomatic gastric sarcoidosis is a difficult diagnosis as it is not only rare but also under-recognized in the majority of cases. Its treatment is exclusively recommended for symptomatic cases only. However, it is of extreme significance to have the asymptomatic patients follow up outpatient regularly to prevent any major complications. Here, we present an interesting case of a 54-year-old African American female patient with only abdominal pain symptoms attributed to a hepatic abscess. A diagnosis of gastric sarcoidosis was solely based on the presence of non-necrotizing granulomas on biopsy following esophagogastroduodenoscopy (EGD). Incidentally, she was also found to have pulmonary sarcoidosis based on imaging. Her abdominal symptoms improved with abscess drainage and so, she was never started on steroids. She was followed up outpatient for pulmonary function tests. The patient continues to do well without any specific treatment for sarcoidosis. This case demonstrates the variability of sarcoidosis and the significance of biopsy in gastric sarcoidosis.

## Introduction

Sarcoidosis is a multi-system disease characterized by non-caseating granuloma. The most common presentation of the disease includes pulmonary and mediastinal involvement. The most commonly involved organs are the lung, liver, and lymph nodes. Gastric involvement is reported in less than 1% of patients presenting with systemic sarcoidosis [1]. Hence, gastric sarcoidosis is a rare entity, and there are only a handful of well-documented histologic pieces of evidence of non-necrotizing granulomas consistent with gastrointestinal (GI) sarcoidosis [2]. The stomach, more specifically the antrum of the stomach, is the most commonly involved area among the entire GI tract [3,4]. The diagnosis of sarcoidosis is supported based on consistent clinical and radiological findings, along with histologic evidence of noncaseating granulomas in the absence of other causative micro-organisms mimicking granulomas. Herein, we present a unique case of abdominal pain due to a sub-hepatic abscess that led to an incidental diagnosis of sarcoidosis involving the GI tract. This case that started with gastric sarcoidosis revealed pulmonary sarcoidosis, demonstrating variable expression of the disease.

## Case presentation

An African American female in her early 50s was admitted to the hospital for worsening abdominal pain. She complained of intermittent generalized abdominal pain that started one month before the presentation. Her pain was not worsened with any movements, not associated with food, and mildly relieved by acetaminophen. She never had similar pain before. She denied any fever, chills, fatigue, cough, shortness of breath, hemoptysis, muscle aches, weight loss, loss of appetite, change in bowel routine, melena or hematochezia, and vomiting. She also denied any skin nodules, rashes, or joint pains. She also denied any recent travels or sick contacts. Her past medical history included mitral valve prolapse. She was a homemaker. She denied any history of smoking, alcohol, or drug abuse. She was not taking any medications at home. She had a strong family history of breast and lung cancer.

**Figure 1:**
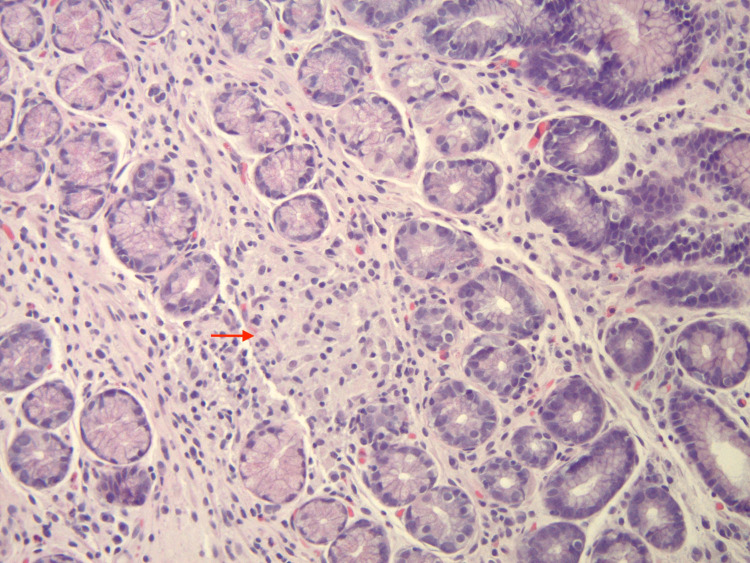
Gastric biopsy shows gastric antral mucosa with intra-mucosal non-caseating granuloma (arrowhead is pointing at the granuloma) (200x, H&E stained).

On the initial presentation, vital signs were normal. Her physical examination was within normal limits except for mild right upper quadrant tenderness without rebound, guarding, or rigidity. There were no signs of lymphadenopathy, leg edema, skin rashes, or nodules. Initial laboratory analysis was significant only for abnormal liver enzymes- aspartate aminotransferase (AST) 67 U/L (normal 13-39 U/L), alkaline phosphatase (ALP) 185 U/L (normal 50-142 U/L), low albumin 3.13 gm/dL (normal 3.5-5.7 gm/dL), and low serum lipase of 6 U/L (normal 11-82 U/L). Serum calcium was 9.5 mg/dL (normal 8.6-10.8 mg/dL). The remainder of the complete blood count and metabolic panel were also normal. Mild polyclonal gammopathy was diagnosed via serum protein electrophoresis. Chest x-ray showed patchy air opacities bilaterally, and COVID-19 polymerase chain reaction (PCR) was negative. Computed tomography (CT) abdomen showed multiple subpleural nodules along with a complicated sub-hepatic cyst/abscess, bowel wall thickening, prominent gastro-hepatic and gastroduodenal lymph nodes. Interventional radiology was consulted for CT-guided pigtail catheter placement to drain a sub-hepatic abscess. Her abdominal pain improved after the sub-hepatic drainage. Fluid analysis was unremarkable for infection or hepatic sarcoidosis. The patient also underwent esophagogastroduodenoscopy (EGD) and colonoscopy for concern of Inflammatory bowel disease based on CT findings, which were grossly normal. The histopathologic results from gastric biopsy-confirmed chronic non-caseating granulomatous gastritis (Figure 1), negative for Helicobacter pylori infection, fungal or mycobacterial stains.

**Figure 2:**
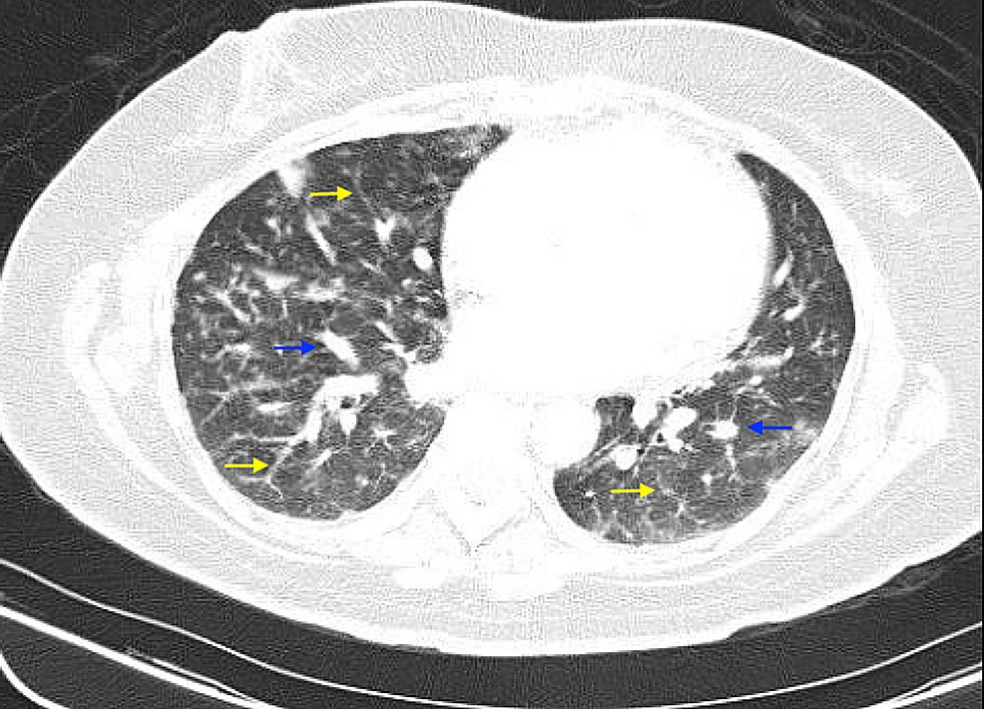
Computed tomography scan with contrast showing bilateral reticulonodular opacities (yellow arrows) and multiple pulmonary nodules (blue arrows).

Subsequent CT-thorax was done, which revealed bilateral reticulonodular opacities and multiple nodules in bilateral lungs (Figure 2). All the infectious workups, including human immunodeficiency virus (HIV), hepatitis A, hepatitis B, hepatitis C, and Quantiferon Gold test for tuberculosis, were negative. Influenza/RSV, group B streptococcus screen, respiratory cultures, fungal cultures, and mycobacterial cultures from sputum were negative. Blood cultures were also negative. Serum beta-D glucan test was negative as well. Tumor markers - CA 19.9 and alpha-fetoprotein were unremarkable. Pulmonology was consulted, and the patient had bronchoscopy with left upper lobe transbronchial biopsy.

**Figure 3:**
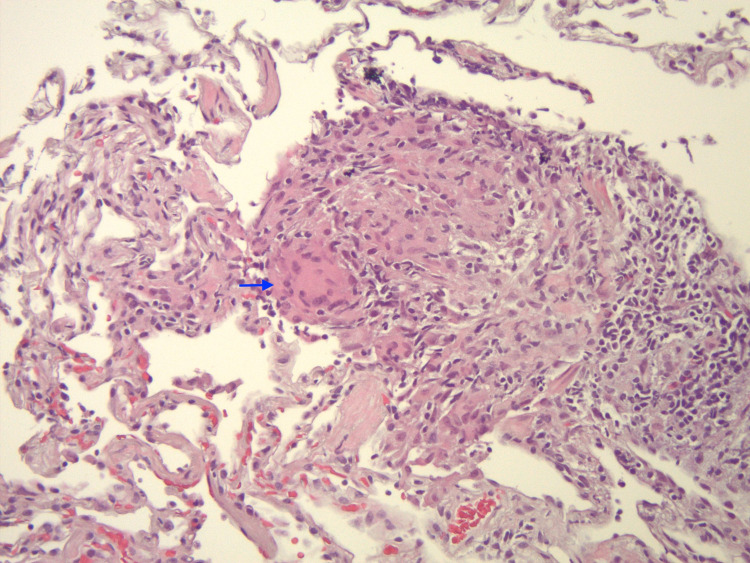
Lung biopsy shows non-caseating granuloma (arrowhead is pointing to the granuloma) (200x, H&E stained).

Based on the laboratory data and clinical findings, a presumed diagnosis of sarcoidosis was made. Since she did not have any specific symptoms like dyspnea, nausea, vomiting, and her abdominal pain had improved after the drainage, she was not started on immunosuppressive agents. She was ready to be discharged. The risks, adverse effects, and benefits of starting Prednisone for the presumed diagnosis of sarcoidosis were discussed with the patient. She elected to wait for lung biopsy results and have a pulmonary function test (PFT) during an outpatient pulmonary clinic visit. The patient did follow up outpatient and had a mild restrictive disease on PFT. The lung biopsy results revealed poorly formed noncaseating granulomas (Figure 3) and intra-alveolar hemorrhage. Stains for fungal and acid-fast organisms were negative. She did not receive any specific therapy as she remained asymptomatic. Repeat PFTs after three months showed significant improvement in the restrictive process.

## Discussion

Gastric sarcoidosis is an uncommon and often asymptomatic condition whose signs and symptoms may resemble other GI diseases. This is responsible for late diagnosis and hence delay in treatment initiation [5]. Gastric sarcoidosis is unique as it can mimic other etiologies of granulomatous gastritis, including Crohn’s disease, Whipple’s disease, hypertrophic gastritis, infections like Helicobacter pylori, syphilis, histoplasmosis, tuberculosis, Langerhans cell histiocytosis, and malignancies like lymphoma and adenocarcinoma [6,7].

Gastric sarcoidosis can present in two different ways, either as diffuse involvement appearing as linitis plastica or as a mucosal ulcer with or without erythema or nodular lesions [8]. Epigastric pain is a common presenting symptom. The presence of pyloric stenosis can also result in nausea and vomiting, with its severity depending on the degree of obstruction. Other symptoms include early satiety, hematemesis, melena, and weight loss [9]. Weight loss can raise clinical suspicion of malignancy. Endoscopic biopsy is considered the gold standard for making a diagnosis of gastric sarcoidosis [5].

The diagnosis of gastric sarcoidosis is challenging to establish in the absence of multisystem involvement. Three components that help to establish the diagnosis of gastric sarcoidosis include noncaseating granulomas on biopsy, exclusion of other granulomatous diseases, and clinical, radiological, or histopathological evidence of involvement of at least one other organ system [10]. Gastric granulomas have coincidentally been reported in up to 10% of patients with pulmonary sarcoidosis. Fluorodeoxyglucose positron emission tomography scanning can be a useful modality in evaluating the disease activity in sarcoidosis [11].

No specific treatment is needed for asymptomatic patients who are diagnosed incidentally. In our case too, since the patient had silent sarcoidosis discovered incidentally, treatment for sarcoidosis was not initiated. But in symptomatic patients, steroids are considered the initial treatment of choice. Proton pump inhibitors can be added to steroids for gastric involvement with ulcers [8]. Surgery might be needed in case of severe gastric outlet obstruction [8].

The pulmonary disease accounts for the majority of sarcoidosis-related deaths [12,13]. The spectrum of pulmonary sarcoidosis ranges from asymptomatic cases with incidentally identified radiological abnormalities to chronic progressive disease with resistance to treatment. Dry cough, chest discomfort, and dyspnea are the most common clinical symptoms of pulmonary sarcoidosis in 30%-50% of patients [14]. Most deaths in sarcoidosis are attributed to respiratory failure. Pulmonary fibrosis, pulmonary hypertension, abnormal pulmonary function, and extensive lung involvement on high-resolution chest CT are considered factors associated with poor prognosis. Steroids are the mainstay of treatment for symptomatic pulmonary sarcoidosis, as with gastric sarcoidosis. Antimetabolites can be used as an alternative in refractory cases or in patients who do not tolerate steroids [15].

Symptomatic hepatic sarcoidosis occurs in only about 5% to 30% of patients [16]. Our patient did not have a fever, pruritis, jaundice, fatigue, nausea, vomiting, weight loss, or hepatomegaly. Liver function test results were not significantly elevated as seen with hepatic sarcoidosis. Abdominal ultrasound and CT scan did not reveal any hepatomegaly or granulomas but showed one 9x7 cm cyst in the left lobe of the liver that was drained. The fluid analysis showed turbid brown fluid with 19,375 RBCs and 363 nucleated cells - 98% neutrophils, no lymphocytes or other cells, and fluid culture was negative. Since hepatic sarcoidosis was very low in differential, liver biopsy was not done.

## Conclusions

This case exemplifies the variable presentation of sarcoidosis. To conclude, physicians should be vigilant about gastric sarcoidosis as a rare presentation. GI sarcoidosis should be considered in patients presenting with recurrent epigastric pain after excluding other causes of granulomatous gastritis. Endoscopy with biopsy should be performed for definitive diagnosis when indicated. Treatment with steroids usually results in remission and provides symptomatic relief. Regular outpatient follow-up is necessary to prevent any serious complications.
